# What is the impact of regulatory guidance and expiry of drug patents on dementia drug prescriptions in England? A trend analysis in the Clinical Practice Research Datalink

**DOI:** 10.1186/s13195-018-0379-6

**Published:** 2018-05-29

**Authors:** Venexia M. Walker, Neil M. Davies, Patrick G. Kehoe, Richard M. Martin

**Affiliations:** 1 Bristol Medical School: Population Health Sciences, University of Bristol, Bristol, UK; 2 MRC Integrative Epidemiology Unit, University of Bristol, Bristol, UK; 3 Dementia Research Group, University of Bristol, Bristol, UK; 4 Bristol Medical School: Translational Health Sciences, University of Bristol, Bristol, UK

**Keywords:** Alzheimer disease, Dementia, Donepezil, Rivastigmine, Galantamine, Memantine, Clinical Practice Research Datalink, National Institute for Health and Care Excellence, Quality and Outcomes Framework, England

## Abstract

**Background:**

Drugs for dementia have been available in England since 1997. Since their launch, there have been several changes to national guidelines and initiatives that may have influenced prescribing. These include changes in National Institute for Health and Care Excellence (NICE) guidance, several government dementia strategies, the addition of dementia to the Quality and Outcomes Framework (QOF), and the expiry of drug patents. Despite this, there has been little research into the effect of these events on prescribing. This paper examines prescribing trends in England using data from the U.K. Clinical Practice Research Datalink since the launch of drugs for dementia up to 1st January 2016.

**Methods:**

We considered the monthly proportion of patients eligible for treatment, with a diagnosis of probable Alzheimer’s disease, receiving their first prescription for each drug class—namely, acetylcholinesterase (AChE) inhibitors (donepezil, rivastigmine, galantamine) and *N*-methyl-d-aspartate (NMDA) receptor antagonists (memantine). Trend analysis using joinpoint models was then applied to identify up to two trend changes per treatment of interest.

**Results:**

The overall trend was for increasing prescriptions in each drug class over the period in which they were studied. This was indicated by the average monthly percentage change, which was 6.0% (95% CI, − 6.4 to 19.9; June 1997 to December 2015) for AChE inhibitors and 15.4% (95% CI, − 77.1 to 480.9; January 2003 to December 2015) for NMDA receptor antagonists. Prescriptions of AChE inhibitors increased at the end of 2012, probably in response to the patent expiry of these drugs earlier that year. The Prime Minister’s Dementia Challenge launched in May 2012 may also have contributed to the observed increase. However, neither this strategy nor patent expiry appeared to influence prescriptions of NMDA receptor antagonists. Instead trend changes in this drug class were driven by NICE guidance released in 2011 that allowed access to these drugs outside of clinical trials.

**Conclusions:**

Dementia drug prescribing does not always respond to factors such as regulatory guidance, recommendations, or patent expiry, and when it does, not necessarily in a predictable way. This suggests that communication with clinicians may need to be improved to use drugs for dementia more cost-effectively.

**Electronic supplementary material:**

The online version of this article (10.1186/s13195-018-0379-6) contains supplementary material, which is available to authorized users.

## Background

There are currently four licensed treatments that provide symptomatic relief for patients with Alzheimer’s disease in England—three acetylcholinesterase (AChE) inhibitors (donepezil, rivastigmine, galantamine) and one *N*-methyl-d-aspartate (NMDA) receptor antagonist (memantine). These drugs are collectively referred to as *drugs for dementia* in the British National Formulary, despite their licensing for Alzheimer’s disease only [1]. Since the first of these drugs became available in 1997, there have been several changes in national guidelines for the treatment of Alzheimer’s disease, as well as several initiatives to encourage better diagnosis and treatment of the disease. Despite this, there has been little research into whether such changes to guidelines and initiatives have directly influenced clinical practice [2, 3]. We examined how prescription rates in England have changed since the launch of these drugs up to 1st January 2016, using data from the U.K. Clinical Practice Research Datalink (CPRD). We investigated how prescribing was affected by changes in National Institute for Health and Care Excellence (NICE) guidance (including the 2006 guidance that was subject to legal challenges), the addition of dementia to the Quality and Outcomes Framework (QOF), the introduction of ambitious government dementia strategies, and the expiry of drug patents. The timing of each of these changes, which may have influenced aspects of drug prescribing and clinical practice, is discussed further below and summarized in Table 1.

**Table 1:** Events prior to 1st January 2016 that potentially affected prescription rates

Event date	Event
May 1997	Donepezil first recorded in CPRD
September 1998	Rivastigmine first recorded in CPRD
January 2001	Galantamine first recorded in CPRD and first NICE guidance released
December 2002	Memantine first recorded in CPRD
November 2006	NICE recommended restricting drug access
September 2007	QOF revised to include dementia
February 2009	First National Dementia Strategy launched
March 2011	NICE removed recommendation restricting drug access
January 2012	Galantamine patent expired
February 2012	Donepezil patent expired
May 2012	Prime Minister’s Dementia Challenge launched
July 2012	Rivastigmine patent expired
April 2014	Memantine patent expired
February 2015	Prime Minister’s Challenge on Dementia 2020 launched

*CPRD* Clinical Practice Research Datalink, *NICE* National Institute for Health and Care Excellence, *QOF* Quality and Outcomes Framework

### NICE guidance on the prescribing of drugs for dementia

In the past NICE guidance has used scores from the Mini Mental State Examination (MMSE), in combination with other measures, to guide whether a patient should be prescribed a drug for dementia. The test, proposed in 1975 by Folstein et al., assesses a patient’s cognition out of a total possible score of 30, where normal cognition is considered as a score of 24 or more [4]. The original NICE guidance, issued in 2001, on the use of drugs to treat Alzheimer’s disease recommended that the three AChE inhibitors should be used for all patients scoring 12 or above on the MMSE until the drugs were deemed no longer effective [5, 6]. In November 2006, NICE revised their guidance so that the use of AChE inhibitors was restricted to patients with moderate Alzheimer’s disease; this was defined as patients scoring between 10 and 20 points on the MMSE. The 2006 guidance was also the first to consider the use of the NMDA receptor antagonist memantine, which was recommended for use only in clinical trials for patients with moderate to severe disease [7]. This revision of the guidance was controversial because of the way in which it assessed cost-effectiveness, which was expected to restrict access to these drugs, and was ultimately the subject of a high court challenge by the Alzheimer’s Society and two drug manufacturers, Eisai and Pfizer [8–10]. This led to a further revision being made to the NICE guidance at the end of March 2011, which recommended AChE inhibitors for patients with mild to moderate Alzheimer’s disease and memantine for patients with moderate to severe Alzheimer’s disease or who could not tolerate AChE inhibitors [11]. For the duration of our present study, treatment had to be initiated by a specialist and deemed effective as long as there has been ‘an improvement or no deterioration in MMSE score, together with evidence of global improvement on the basis of behavioral and/or functional assessment’ [6].

### Inclusion of dementia on the QOF

QOF is a voluntary incentive program, introduced in 2004, to improve services in primary care [12]. Dementia first appeared in QOF as an ‘indicator’ in September 2007 [13]. There are currently three indicators for dementia included in the framework. The first requires that the practice establish and maintain a register of patients diagnosed with dementia, and the other two indicators refer to the ongoing management of the disease [14]. The inclusion of dementia on the QOF could therefore have encouraged a greater focus on the diagnosis and pharmacological management of the disease in participating practices.

### Government dementia strategies

The first National Dementia Strategy was launched by the Department of Health in February 2009. The aim of that strategy was ‘to ensure that significant improvements are made to dementia services across three key areas: improved awareness, earlier diagnosis and intervention, and a higher quality of care’ [15]. This strategy was followed in 2012 by the Prime Minister’s Dementia Challenge, which looked to improve care and research by 2015, and more recently by the Prime Minister’s Challenge on Dementia 2020 [16, 17]. The most recent strategy aims to build on the work of its predecessors to make England the best place for both dementia care and research. In general such strategies may help to increase the awareness of dementia for both the public and health services [18, 19].

### Drug patents

The King’s Fund charity found that the prescription of generic drugs over their patented alternatives has ‘saved the NHS around £7.1 billion and allowed more than 490 million more items to be prescribed to patients’ between 1976 and 2013 [20]. AChE inhibitors for the treatment of Alzheimer’s disease became available generically from 2012, whereas NMDA receptor antagonists became available generically from 2014 (Table 2) [21]. Therefore, in recent years the cost of drugs for dementia has decreased significantly from previous years. This serves as a potential factor in rates of prescribing, particularly in publicly funded health care services such as the NHS in England.

**Table 2:** Patent information for the drugs used for dementia [21]

Generic name	Patent name (manufacturer)	Drug class	Patent expiry
Donepezil	Aricept (Eisai / Pfizer)	AChE inhibitor	January 2012
Rivastigmine	Exelon (Novartis)	AChE inhibitor	February 2012
Galantamine	Reminyl (Shire)	AChE inhibitor	July 2012
Memantine	Ebixa (Lundbeck)	NMDA receptor antagonist	April 2014

*AChE* Acetylcholinesterase, *NMDA N*-methyl-d-aspartate

## Methods

### Aim

The aim of the study was to examine prescribing trends in England from the launch of the drugs for dementia up to 1st January 2016, using data from the CPRD.

### Design

**Fig. 1:**
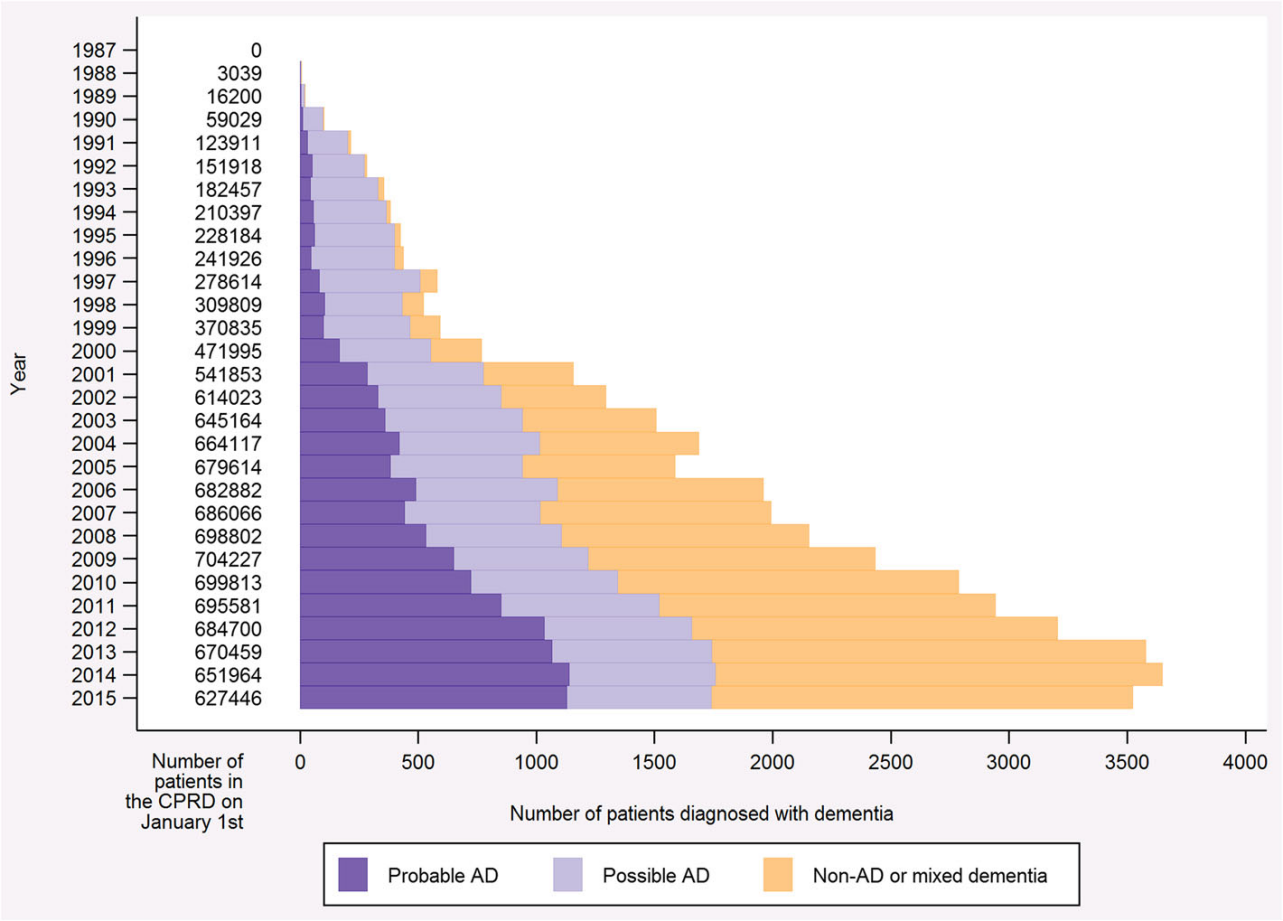
Bar graph illustrating the number of patients diagnosed with dementia, by diagnosis type. The data presented are restricted to patients who received a diagnosis prior to 1st January 2016 and are from an English practice with a last data collection date in 2016 to reflect the main analysis. Definitions for each of the diagnoses are presented in Table 3.

This study was a joinpoint analysis of the proportion of patients eligible for treatment, with a diagnosis of ‘probable Alzheimer’s disease’, receiving their first prescription for the treatment of interest in each month. We defined patients as eligible for their first prescription if they had the diagnosis of interest with no previous prescription for the treatment of interest. The time period was measured in units of 1 month because this was the smallest clinically meaningful measure we could realistically define. We investigated treatment rates as a proportion of eligible patients because the underlying rate of diagnosis of Alzheimer’s disease, as well as non-Alzheimer’s disease and mixed dementias, has changed over time in the CPRD (Fig. 1). The joinpoint analysis used in this study has been developed for incidence rates, so prevalent drug use, which requires consideration of both incidence and continued drug use, was not studied.

The four drugs for dementia were separated according to drug class (i.e., AChE inhibitors and NMDA receptor antagonists). Exposure date was taken to be the date on which the first prescription requesting the drug(s) being considered was recorded. This allowed patients who had previously been prescribed AChE inhibitors to be included in the NMDA receptor antagonist analysis. This is necessary because NMDA receptor antagonists may be prescribed alongside AChE inhibitors and are often given to patients later in the course of their disease, potentially following exposure to AChE inhibitors.

### Setting

In this study we used data from the CPRD, an ongoing U.K.-based primary care database established in 1987. The data used in this study were obtained as part of a larger project investigating whether commonly prescribed drugs can be repurposed for the prevention or treatment of Alzheimer’s and other neurodegenerative diseases [22]. For this project, we sampled patients older than 40 years of age with at least 12 consecutive months of records classified as ‘acceptable’ by the CPRD from an ‘up to standard’ practice. The data were taken from the March 2016 CPRD GOLD database snapshot, which covered the period from 1st January 1987 to 29th February 2016, inclusive.

### Sample

For this study, we considered the data available from 1st January 1987 to 31st December 2015, inclusive, from practices with a last data collection date in 2016; this ensured that all data were complete for the time frame being considered. We also restricted the data to English practices. This is because guidelines and initiatives can differ by nation in the United Kingdom; for example, all nations are subject to patent expiry, but the National Dementia Strategy is applicable only to England, with other nations having their own strategies. Additional file 1 presents a sensitivity analysis investigating the effect of limiting the study to practices in England. The analysis concludes that because the majority of the CPRD data is obtained from English practices and the proportion of people included in the study is similar for England and the CPRD as whole, the representativeness of the CPRD is likely to have been preserved. To be included in the study, a patient had to have a diagnosis of dementia as determined by a read or product code (*see* reference [23] for code lists). Read and product codes uniquely identify clinical terms and prescriptions, respectively, in the CPRD and are recorded by the general practitioner at the time of the consultation with the date [24]. The validity of codes for dementia diagnoses in the CPRD has previously been studied and was found to be in concordance with depersonalized written records relating to the diagnosis [25]. The diagnoses and their definitions as used in the present study are provided in Table 3. We used treatment to define diagnosis under the assumption that treatment implies diagnosis. Diagnosis date was taken to be the first date on which a code from any of the lists was recorded. We performed a sensitivity analysis to test the diagnosis definitions, which is presented in full in Additional file 2. The analysis considered the sensitivity and specificity of the diagnoses in the CPRD dataset using linked data from the Office of National Statistics (ONS) death registry and the Hospital Episode Statistics (HES) inpatient dataset. We found there to be high specificity (HES, 62.9–79.1%; ONS, 57.5–75.1%) and variable sensitivity (HES, 37.3–71.6%; ONS, 36.0–80.4%). The high specificity demonstrated in this analysis reflects our conservative approach when constructing the code lists. Consequently, we expected a lower sensitivity, and this is in line with what we observed.

**Table 3:** Diagnosis definitions used in the study, presented with the number of patients

Diagnosis	Definition	Patients
Probable AD	Patients with one or more codes on the list ‘probable AD’. Patients may also have codes on the lists ‘possible AD’, ‘donepezil’, ‘rivastigmine’, ‘galantamine’ and ‘memantine’.	10,651
Possible AD	Patients with one or more codes on the list ‘possible AD’. Patients may also have codes on the lists ‘donepezil’, ‘rivastigmine’, ‘galantamine’ and ‘memantine’.	12,167
Non-AD and mixed dementias	Patients with one or more codes on any of the following lists: ‘probable AD’, ‘possible AD’, ‘other dementia’, ‘vascular dementia’, ‘non-specific dementia’, ‘donepezil’, ‘rivastigmine’, ‘galantamine’ and ‘memantine’, who do not meet the above criteria.	17,384

*AD* Alzheimer’s disease

The data presented are restricted to patients who received a diagnosis prior to 1st January 2016 and are from an English practice with a last data collection date in 2016 to reflect the main analysis. The total number of patients with ‘any dementia’ is 40,202

### Analysis

The analysis of each treatment of interest started on the first day of the month following the first recorded prescription for that treatment. For example, the first prescription for NMDA receptor antagonists occurred on 16th December 2002, so the analysis of this drug class started on 1st January 2003. For each patient, we used the month and year of diagnosis (Table 3) and first prescription. For each month, we calculated the following: (A) the number of patients receiving their first prescription in that month and (B) the number of patients with a diagnosis who had not received treatment before the first of the month. Dividing A by B provided the proportion of patients with diagnoses who received their first prescription for the treatment of interest each month. We also calculated the SE of this proportion [26]. Trend analysis using joinpoint models was then conducted. The optimal number of joinpoints, as determined by the software and up to a maximum number of 2, was used to select the model. We refer to the period between two joinpoints as a ‘segment’ and number them chronologically. Our model assumes that the rate of prescription ‘changes at a constant percentage of the rate of the previous year’ [27] and so is determined by the following equation: ln*y* = *xb*. This allows us to consider the monthly percent change. The trend over the entire study period is summarized using the average monthly percent change. This is calculated as the average of the monthly percent changes, weighted by segment length [28]. All analysis was conducted using Joinpoint Regression Program (version 4.3.1.0; National Cancer Institute, Bethesda, MD, USA) and Stata (version 14.1; StataCorp, College Station, TX, USA) software [29, 30]. The model specifications for the joinpoint analyses are the software’s default with dependent variable type set to ‘proportion’ and the maximum number of join points set to 2. The Stata code used in this analysis is available from GitHub (https://github.com/venexia/DementiaDrugsCPRD) [31].

### News search

Several of the national guidelines and initiatives considered in this study may have increased awareness of dementia, including Alzheimer’s disease. To investigate this, we downloaded the Google Trends (https://trends.google.com/trends/) data for news searches in England for the disease term ‘Alzheimer’s disease’ from 1st January 2008 up to 1st January 2016 [32]. Unfortunately, data were not recorded prior to 2008, so we cannot comment on the effect media coverage may have had on trend changes identified before this point in time. As with the main analysis, data were processed and plotted using Stata (version 14.1; StataCorp, College Station, TX, USA), and the code is available from GitHub (https://github.com/venexia/DementiaDrugsCPRD) [29, 30].

## Results

### Trend analysis for AChE inhibitors

**Fig. 2:**
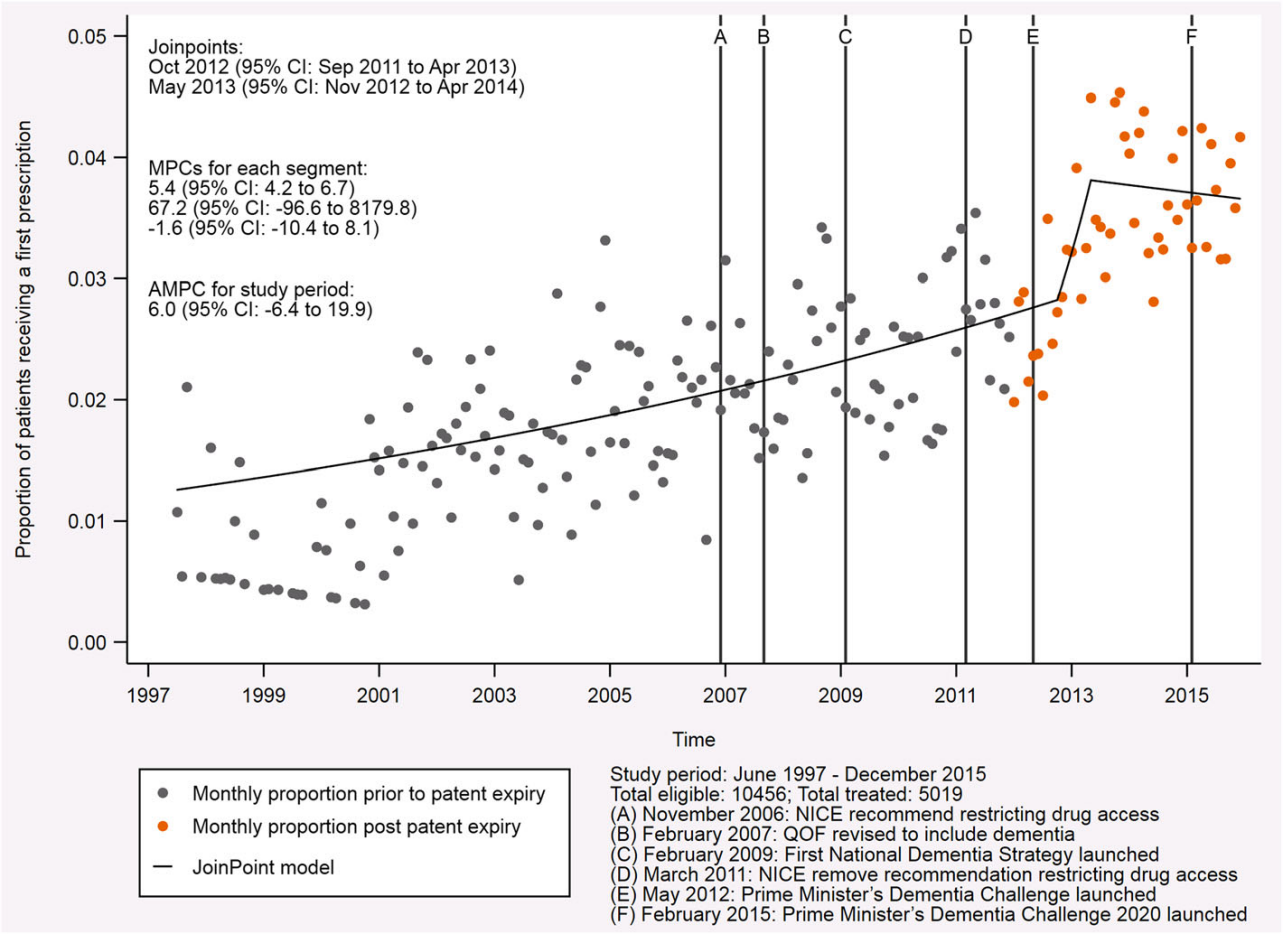
Indicative graph of acetylcholinesterase (AChE) inhibitor prescriptions in patients with probable Alzheimer’s disease. This graph shows the proportion of patients with probable Alzheimer’s disease receiving their first prescription for an AChE inhibitor each month from June 1997 to December 2015. The fixed lines indicate events with the potential to affect prescription rates during the study period. The joinpoints, monthly percent change (MPC) for each segment, and the average monthly percent change (AMPC) for the entire study period are also presented

The proportion of patients with probable Alzheimer’s disease receiving their first prescription for an AChE inhibitor increased throughout the study period (Fig. 2). This is reflected in the average monthly percent change, which was 6.0 (95% CI, − 6.4 to 19.9) for the period from June 1997 to December 2015. For much of the study period, the trend was for an increasing proportion of patients to receive their first prescription for an AChE inhibitor with a monthly percent change of 5.4 (95% CI, 4.2 to 6.7). In October 2012 (95% CI; September 2011 to April 2013; *p* = 0.816), the prescription rate surged with a monthly percent change of 67.2 (95% CI, − 96.6 to 8179.8). Less than 1 year later, in May 2013 (95% CI; November 2012 to April 2014; *p* = 0.789), the trend reversed so that prescription rates were falling. In the months that followed, the monthly percent change had a value of − 1.6 (95% CI, − 10.4 to 8.1), falling below zero for the first time since the launch of these drugs.

### Trend analysis for NMDA receptor antagonists

**Fig. 3:**
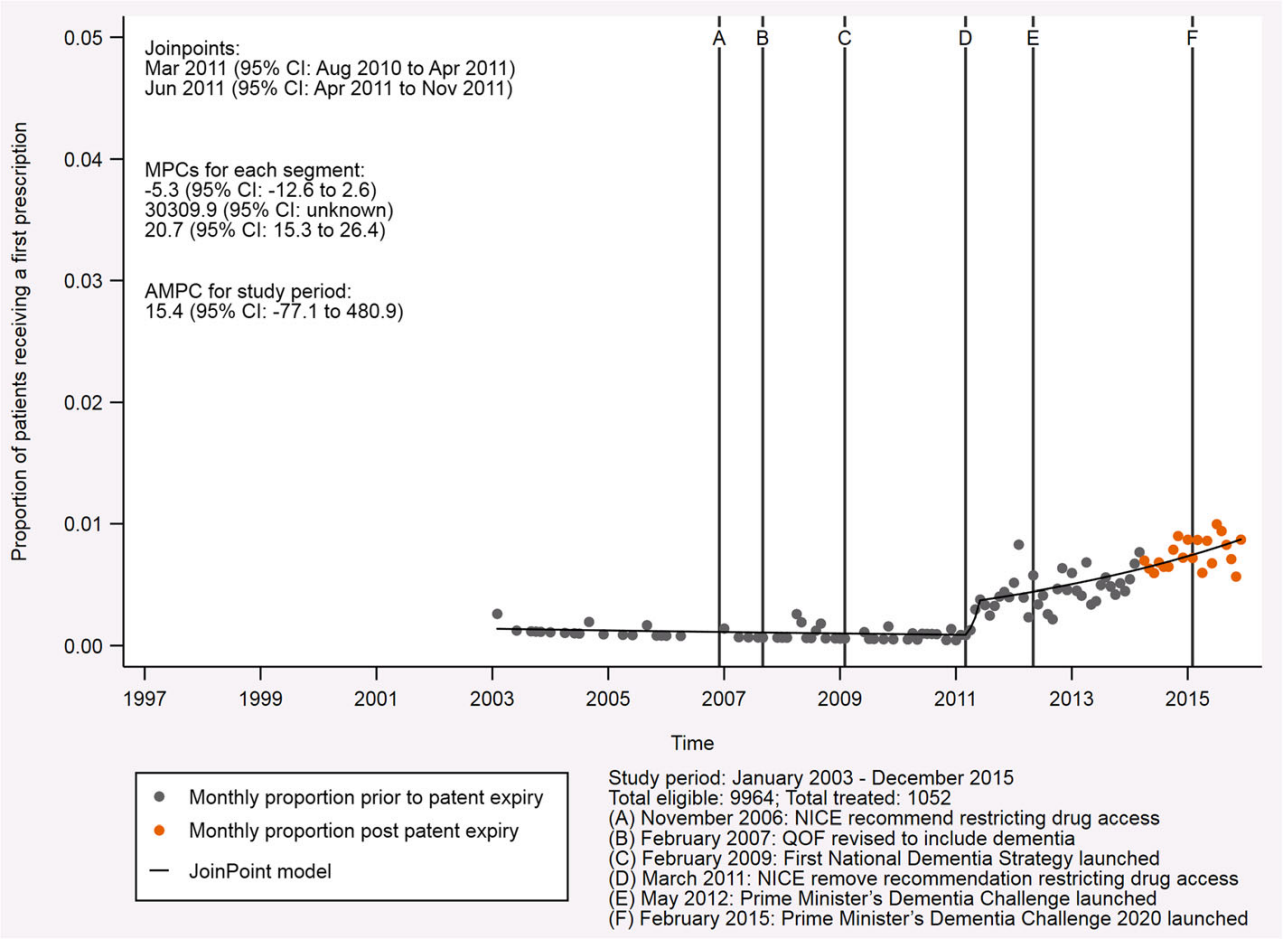
Indicative graph of *N*-methyl-d-aspartate (NMDA) receptor antagonist prescriptions in patients with probable Alzheimer’s disease. This graph shows the proportion of patients with probable Alzheimer’s disease receiving their first prescription for an NMDA receptor antagonist each month from January 2003 to December 2015. The fixed lines indicate events with the potential to affect prescription rates during the study period. The joinpoints, the monthly percent change (MPC) for each segment, and the average monthly percent change (AMPC) for the study period are also presented

Figure 3 presents the equivalent analysis for the NMDA receptor antagonist memantine. Memantine became available in January 2003 and was prescribed much less often than the other drugs, despite similar numbers of eligible patients. This is partly related to the indication of these drugs. Memantine is generally recommended for more advanced disease than the AChE inhibitors and is often added to a prescription of AChE inhibitors following progression of the disease. Despite this, as observed for the AChE inhibitors, the proportion of patients with probable Alzheimer’s disease receiving their first prescription for an NMDA receptor antagonist increased on average throughout the study period. The average monthly percent change for the period from January 2003 to December 2015 was 15.4 (95% CI, − 77.1 to 480.9), though the 95% CI around this estimate is large. The initial trend for prescribing of this drug showed a reduced number of prescriptions in the time that followed the launch with a monthly percent change of − 5.3 (95% CI, − 12.6 to 2.6). This changed around March 2011 (95% CI, August 2010 to April 2011, *p* = 0.892) to a very strong trend for increased prescribing. From the second trend change in June 2011 (95% CI, April 2011 to November 2011, *p* = 0.896) until the end of the study in December 2015, this trend reduced to a monthly percent change of 20.7 (95% CI, 15.3 to 26.4). This indicates a continuing increase in the prescriptions for NMDA receptor antagonists in recent years, albeit substantially reduced from the rise observed between March and June 2011. The complete output for both this analysis and that relating to AChE inhibitors is provided in Additional file 3.

### Sensitivity analyses

We repeated the main analysis, which considers the diagnosis ‘probable Alzheimer’s disease’, with relaxed diagnosis definitions to test the sensitivity of our results. We did this in two ways: (1) introducing codes that represented what may be lesser degrees of confidence in the accuracy of Alzheimer’s disease diagnosis (termed *any Alzheimer’s disease*) and (2) introducing codes capturing other types of dementia (termed *any dementia*). The results of these further analyses are provided in Additional file 3, and a summary of all results can be found in Table 4. For NMDA receptor antagonists, the joinpoint analysis is consistent regardless of the diagnosis definition used. However, for AChE inhibitors, the joinpoint analysis varies according to the diagnosis definition used, though the two sensitivity analyses are reasonably consistent with each other.

**Table 4:** Comparison of the sample sizes and joinpoint estimates, presented with 95% confidence intervals, for all analyses

	Probable AD	Any AD	Any dementia
Source	Main analysis	Additional file 3	Additional file 3
Diagnoses	Probable AD	Probable AD Possible AD	Probable AD Possible AD Non-AD and mixed dementias
AChE inhibitors	Eligible: 10,456 Treated: 5019 Joinpoint 1: Oct 2012 (Sep 2011–Apr 2013) Joinpoint 2: May 2013 (Nov 2012–Apr 2014)	Eligible: 21,342 Treated: 6449 Joinpoint 1: Jun 1999 (Apr 1998–Dec 2000) Joinpoint 2: Jun 2001 (Sep 2000–Mar 2002)	Eligible: 38,650 Treated: 9896 Joinpoint 1: Aug 2000 (Jun 1998–Nov 2000) Joinpoint 2: Jan 2001 (Sep 2000–Nov 2001)
NMDA receptor antagonists	Eligible: 9964 Treated: 1052 Joinpoint 1: Mar 2011 (Aug 2010–Apr 2011) Joinpoint 2: Jun 2011 (Apr 2011–Nov 2011)	Eligible: 18,930 Treated: 1309 Joinpoint 1: Sep 2010 (Dec 2009–Apr 2011) Joinpoint 2: Nov 2011 (Apr 2011–Mar 2012)	Eligible: 35,625 Treated: 1961 Joinpoint 1: Aug 2010 (Nov 2009–Dec 2010) Joinpoint 2: Nov 2011 (Aug 2011–Mar 2012)

*AChE* Acetylcholinesterase, *AD* Alzheimer’s disease, *NMDA N*-methyl-d-aspartate

### News search

**Fig. 4:**
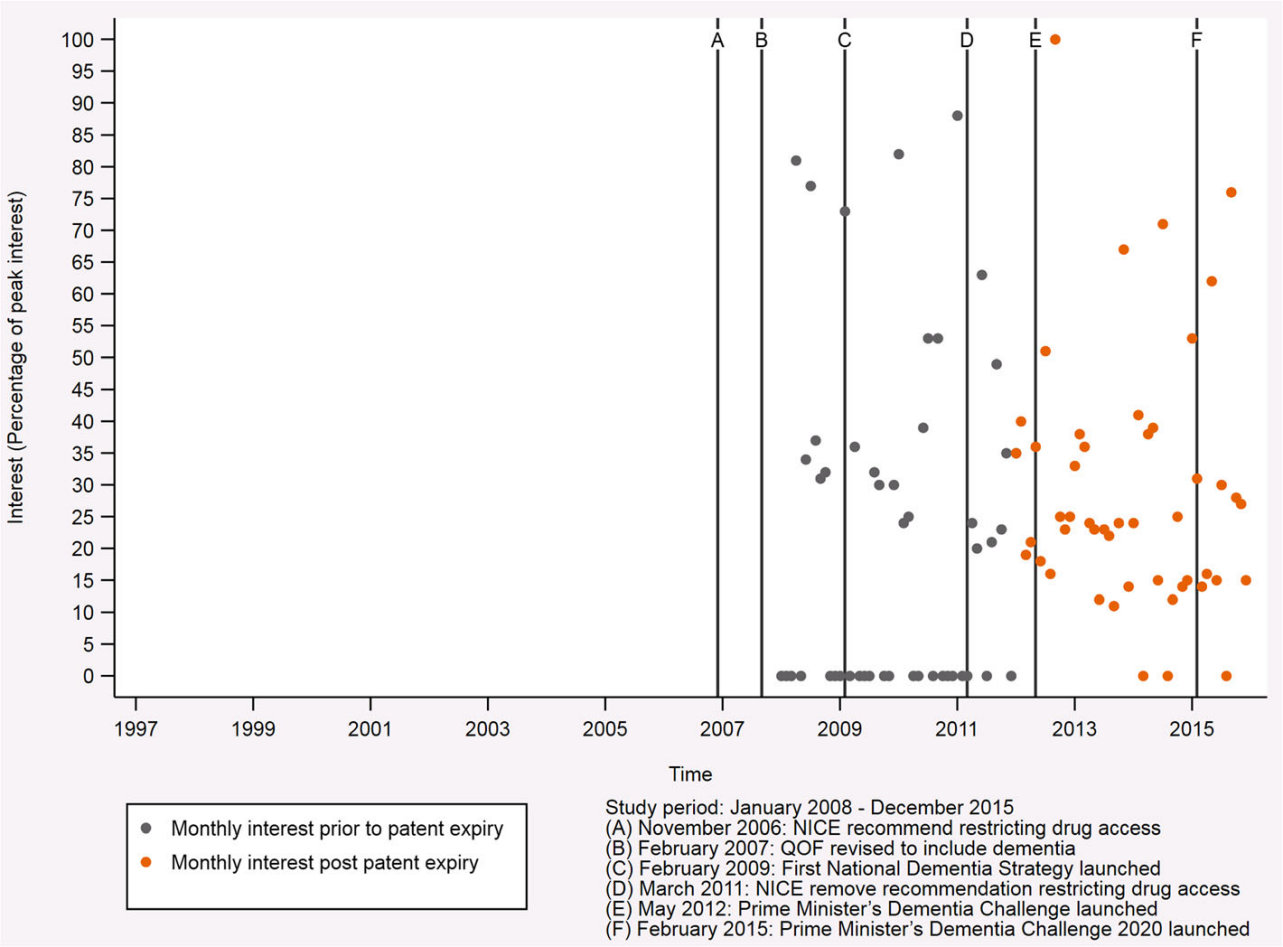
Google Trends data for news searches in England for the disease term ‘Alzheimer’s disease’. This graph shows the interest in the disease term ‘Alzheimer’s disease’ each month from January 2008 to December 2015, inclusive. Interest is given as a percentage scaled against peak popularity, which is represented as a value of 100% and occurred for the downloaded data in September 2012. Values of zero indicate insufficient data for that month

Figure 4 presents the Google Trends data for news searches in England for the disease term ‘Alzheimer’s disease’ each month from January 2008 to December 2015, inclusive. There were no strong trends in the interest for the search term, with values indicating both low and high interest occurring throughout the period studied. Months with insufficient data, indicating little interest in the search term, became less common over the period studied, with the most recent occurring in August 2015. Interest peaked in September 2012 and was also high in January 2011 (88%), January 2010 (82%) and April 2008 (81%).

## Discussion

The first trend change for the proportion of patients with probable Alzheimer’s disease receiving their first prescription for an AChE inhibitor occurred in October 2012 (95% CI, September 2011 to April 2013, *p* = 0.816). At this time, a long-term steadily increasing trend became a very strong increasing trend. This surge could be related to two factors. Firstly, the patents expired on the three drugs in this class in 2012—galantamine in January 2012, donepezil in February 2012 and rivastigmine in July 2012. Secondly, the Prime Minister’s Dementia Challenge launched in May 2012. It is likely that the reduction in cost of these drugs, which resulted from their patents expiring, in combination with increased awareness of dementia due to the Prime Minister’s Dementia Challenge, led to this substantial change in prescription rates we observed. In addition to these factors, a large amount of literature concerning AChE inhibitors had been published ahead of the revisions to the NICE guidance in 2011. Although this is unlikely to have caused the sharp surge that we observed, it could have contributed to the long-term steadily increasing trend observed prior to this change. A systematic review which covers the literature through November 2014 (i.e., after all join points identified in our analysis but 13 months before the end of our study) summarizes the literature available at that time [33]. It shows that several studies published between 2003 and 2008 suggested that patients with mild to moderate Alzheimer’s disease could benefit from AChE inhibitors with estimated ‘improvements on the order of 1.5 MMSE (30-point scale)’. We therefore cannot rule out a potential effect of the literature on prescribing, even though the authors of the review questioned whether such an improvement was clinically meaningful when all the evidence was presented together. Further to the support from the literature, the Google Trends data for news searches in England also suggested increased awareness around the time of this trend change. The interest for the search term ‘Alzheimer’s disease’ was at its maximum in September 2012 (based on the data available from January 2008 to December 2015, inclusive), which could indicate interest among the public.

The second trend change in the AChE inhibitor analysis occurred in May 2013 (95% CI, November 2012 to April 2014, *p* = 0.789), less than 1 year after the initial change for this drug class and with overlapping 95% CIs. This change signals the end of the surge in prescribing and the start of a decreasing trend in prescriptions. This is not unexpected, because patent expiry may have led to a form of ‘catch-up prescribing’ whereby people who were previously denied access to the drug were granted access at this time owing to its newly reduced cost. This would result in the apparent decreasing trend once ‘catch-up prescribing’ was complete, which is suggested by the trend analysis but is not as clear when considering the raw data points. These results differ from the sensitivity analyses that considered relaxed diagnosis definitions, though the ‘any Alzheimer’s disease’ and ‘any dementia’ analyses were in line with each other. This suggests that prescribing for patients with probable Alzheimer’s disease was more consistent, as one might expect, across the study than for other groups. This could indicate that patients with dementias other than probable Alzheimer’s disease (i.e., with unlicensed indications) were receiving these drugs and that their prescriptions were subject to change over the period studied. Further to this, large increases in prescriptions are observed as the diagnosis definition is relaxed. This could provide further evidence for the possible unlicensed use of this drug class. The literature at that time also reflects ongoing discussion concerning the benefit of these drugs for indications other than Alzheimer’s disease. For example, a 2012 review by Rodda and Carter discusses their use in vascular dementia, dementia with Lewy bodies and Parkinson’s disease dementia [34]. Alternatively, it could be attributed to the fluctuating course of symptoms that some people with dementia experience or increased recognition of mixed diagnoses where there is evidence of Alzheimer’s disease in addition to other forms of dementia, both of which might lead to treatment changes.

The trend changes in the NMDA receptor antagonist analysis occurred in March 2011 (95% CI, August 2010 to April 2011, *p* = 0.891) and June 2011 (95% CI, April 2011 to November 2011, *p* = 0.896). Notably, the 95% CI for the first trend change ends in April 2011, which is when the 95% CI for the second trend change begins. This suggests that the trend changes may be related. The first of these trend changes marks the start of a strong increasing trend that changes to a steadily increasing trend following the second trend change. In March 2011 NICE introduced guidelines that recommended the prescription of memantine for patients with moderate to severe Alzheimer’s disease or for those people who could not tolerate AChE inhibitors. This replaced existing guidelines that restricted access to memantine to patients participating in clinical trials. It would therefore seem that these trend changes relate to the transition between the existing guidelines and those introduced in March 2011. In addition, we observed the second highest peak in interest (88% of maximum interest) for the disease term ‘Alzheimer’s disease’ in the Google Trends data for news searches in England in January 2011. In this month, the ‘Final Appraisal Determination on Donepezil, galantamine, rivastigmine and memantine for the treatment of Alzheimer’s disease’ was released. NICE defined this document as ‘the appraisal committee’s final draft guidance about using a treatment or group of treatments in the NHS’, which becomes guidance if not appealed [35]. The increase in news searches around this time, and its alignment with the release of the final draft guidance, supports the idea of a transition in prescribing practice due to the NICE guidance. Finally, the evidence concerning the use of memantine is summarized in a technology appraisal conducted by NICE in 2011 to support their guidance [11]. We cannot disentangle the role that this information from several studies published prior to the trend change might have played in changes to prescribing.

Interestingly, neither of the trend changes in the NMDA receptor antagonist analysis aligns with those observed for the AChE inhibitors. This suggests that the NICE guidelines, which were implemented at the same time for both drug classes, may not have been as effective for AChE inhibitors. This is likely due to the fact that these drugs were available outside of clinical trials prior to the restrictive guidelines recommended in 2006. The sensitivity analyses conducted for the NMDA receptor antagonists were consistent with these results, regardless of the diagnosis definition used. The first of the joinpoints for all NMDA receptor antagonist analyses occurred in the 7-month period between August 2010 and March 2011, and the second occurred in the 6-month period between June 2011 and November 2011. This high level of consistency across diagnosis definitions indicates a clear pattern in prescribing, suggestive of a distinct change in practice. This provides additional support for our inferences concerning the impact of the 2011 NICE guidance on the NMDA receptor antagonist drug class.

### Strengths and limitations

The key strength of this study is the large sample of primary care data with prescribing information, provided by the CPRD. The CPRD is ‘broadly representative of the UK general population’ and was generally comparable to the last census in 2011 for age, sex and ethnicity despite young people and smaller practices tending to be slightly underrepresented [36, 37]. Our data extract contains 40,202 patients diagnosed with dementia in England up to 1st January 2016 (note that data are restricted to practices with a last data collection date in 2016), including 10,651 with probable Alzheimer’s disease and a further 12,167 with possible Alzheimer’s disease. A further strength of our study is the long follow-up of patients that allowed us to consider patients who did not receive immediate treatment. This is important because pharmacological interventions for Alzheimer’s disease have historically considered severity as part of the prescribing decision, so there is likely to be a treatment delay after initial diagnosis for those presenting with mild disease.

The main limitation of our study is the likelihood of missed diagnoses. This is demonstrated within our dataset, because there were 1231 patients receiving one of the treatments of interest who did not have any form of recorded dementia diagnosis. Missed diagnoses are likely to be due to (1) outdated or non-specific diagnoses (i.e., type of dementia is not updated once established), (2) diagnoses received outside of primary care (i.e., from a specialist service) and (3) unrecorded diagnoses in primary care (i.e., a diagnosis is given but not added to a record). Missed diagnoses have been explored in sensitivity analyses by testing the sensitivity and specificity of our diagnosis definitions (Additional file 2) and by relaxing the diagnosis definition from ‘probable Alzheimer’s disease’ to include other less certain codes for the disease and other types of dementia (Additional file 3). Neither of these sensitivity analyses provided any cause for concern. A final limitation of this study is the difficulty in determining the lag time between an event and a trend change to assess the impact of the event. To allow for this, we have focused on events that are considered to be of greatest impact—for example, changes at a national level—and so we expect any effect associated with them to be evident if present. However, this prevents us from covering all the factors that may influence prescriptions for drugs for dementia during the study period; for example, we cannot comment on all papers concerning these drugs published during this time.

## Conclusions

Analysis of both drug classes indicates that inclusion of dementia in QOF had no effect on prescribing trends and the other factors had mixed effects. NICE guidance on the prescribing of drugs for dementia aligned with trend changes for NMDA receptor antagonists but not AChE inhibitors. The guidance that had the noticeable effect was released in March 2011 and allowed the NMDA receptor antagonist memantine to be used outside of clinical trials. All other guidance for both this drug and AChE inhibitors, including that which recommended restricting access, did not align with trend changes. Government dementia strategies also appear to have had mixed results, with the Prime Minister’s Dementia Challenge (launched May 2012) being the only strategy to align with a trend change. Although this strategy is likely to have increased awareness of dementia around the time of the October 2012 trend change for AChE inhibitors, we believe that the more likely cause of this change is the patent expiry of the drugs in this class. This will have reduced the cost of these drugs and potentially led to a surge in prescribing, such as that observed in our trend analysis. The events considered here highlight the many factors that may have influenced prescribing rates and the challenges in assessing the impact of a given event. Overall it would seem that the proportion of patients receiving prescriptions increased over the period studied, regardless of changing guidelines and other initiatives. Furthermore, given the increase in diagnoses of dementia and, more specifically, Alzheimer’s disease reported in the CPRD (Fig. 1), the absolute number of prescriptions has increased considerably over the period studied.

To our knowledge, there are two other studies that have considered prescribing trends, and these were focused mainly on the impact of the National Dementia Strategy [2, 3]. Our study extends the findings of these previous studies because it considers trends since the launch of these drugs and implements a joinpoint model as a hypothesis-free approach for the factors affecting prescribing. We have observed that prescription rates in England do not always respond to factors such as regulatory guidance, recommendations or patent expiry, and when they do, not necessarily in a predictable way. This suggests that communication with clinicians may need to be improved to use drugs for dementia more cost-effectively. In addition to this, the present study provides insight into the factors that may have influenced prescription rates of drugs for dementia in England since their launch in 1997. This is essential for accurate assessment of the effectiveness of these treatments and to adjust for them in other forms of analyses, particularly as factors that may modify the rates of disease progression. This study may also help to inform the handling of regulatory guidance and recommendations concerning drugs for dementia in the future.

## Additional files

Additional file 1: Analysis of the representativeness of the CPRD. (PDF 343 kb)

Additional file 2: Analysis of the sensitivity and specificity of the diagnoses. (PDF 438 kb)

Additional file 3: Analysis using alternative diagnoses. (PDF 1728 kb)

## Abbreviations

AChE: Acetylcholinesterase
AD: Alzheimer’s disease
AMPC: Average monthly percent change
CPRD: Clinical Practice Research Datalink
HES: Hospital Episode Statistics
MMSE: Mini Mental State Examination
MPC: Monthly percent change
NICE: National Institute for Health and Care Excellence
NMDA: *N*-methyl-d-aspartate
ONS: Office of National Statistics
QOF: Quality and Outcomes Framework
