# Supplementary material for: What is the impact of regulatory guidance and expiry of drug patents on dementia drug prescriptions in England? A trend analysis in the Clinical Practice Research Datalink
Source: Alzheimers Res Ther. 2018 May 29;10:51. doi: 10.1186/s13195-018-0379-6 (PMC5975499; doi:10.1186/s13195-018-0379-6)
Supplement: Supplementary file 1 — Analysis of the representativeness of the CPRD. (PDF 343 kb) [file 13195_2018_379_MOESM1_ESM.pdf]

Additional file 1

Analysis of the representativeness of the CPRD

The CPRD is known to be broadly representative of the UK population. (1,2) The aim of these sensitivity analyses is therefore to determine the effect of our study criteria, especially the exclusion of non-English data, on this representativeness. There is a total of 135,144 patients from 675 practices that have a diagnosis of dementia recorded in the CPRD according to our definition. Here we present the proportion of those patients and practices that meet the criteria of our study for each nation in the UK. We will also present the distribution of patients and practices included in our study at the regional level. This information will allow us to assess whether the representativeness of the CPRD has been maintained in our study.

Table S1.1: The distribution of patients that meet the criteria of our study by nation.

Nation	Study	CPRD	Percentage
England	40202	103595	38.8
Northern Ireland	4380	5146	85.1
Scotland	11114	15214	73.1
Wales	8090	11159	72.5
**Total**	**63786**	**135114**	**47.2**

Table S1.2: The distribution of practices that meet the criteria of our study by nation.

Nation	Study	CPRD	Percentage
England	195	522	37.4
Northern Ireland	21	22	95.5
Scotland	67	79	84.8
Wales	44	52	84.6
**Total**	**327**	**675**	**48.4**

Tables S1.1 and S1.2 show the distribution of patients and practices at the national level. This information should help to understand the effect of excluding non-English practices from our study. Out of the 135,144 patients recorded as having dementia in the CPRD, 47.2% of patients qualify for our study. In England, this percentage is slightly lower at 38.8% of patients. Given the similarity of these percentages and the fact that 76.7% of patients in the CPRD are registered at an English practice, the English population is likely to be representative of the CPRD dataset as a whole. Table S1.2 confirms our observations from Table S1.1 with the distribution of practices mostly in line with the distribution of patients. We see a slight decrease in the percentage of English practices qualifying for the study with a slight increase in all other nations. The difference in direction of these changes means there is little overall change in the UK percentage, providing further evidence for the English practices being representative of the CPRD as a whole. It is worth noting that the percentage of patients qualifying for our study in other nations of the UK is much higher than in England. This is due to the other criteria on our study, namely that practices should have their last data collected in 2016. While many practices outside of England met this criterion, data collection is less consistent in England. This is discussed in further detail below.

Table S1.3: The distribution of patients that meet the criteria of our study by region.

Region	Study	CPRD	Percentage
North East	504	2365	21.3
North West	6383	15914	40.1
Yorkshire and the Humber	561	5109	11.0
East Midlands	0	4574	0.0
West Midlands	3731	11552	32.3
East of England	1975	10435	18.9
South West	4718	12587	37.5
South Central	7659	16041	47.7
London	5544	11817	46.9
South East Coast	9127	13201	69.1
**Total**	**40202**	**103595**	**38.8**

Table S1.4: The distribution of practices that meet the criteria of our study by region.

Region	Study	CPRD	Percentage
North East	3	12	25.0
North West	29	80	36.3
Yorkshire and the Humber	3	29	10.3
East Midlands	0	25	0.0
West Midlands	20	58	34.5
East of England	9	52	17.3
South West	19	60	31.7
South Central	28	54	51.9
London	37	87	42.5
South East Coast	47	65	72.3
**Total**	**195**	**522**	**37.4**

Tables S1.3 and S1.4 show the distribution of patients and practices within England at the regional level. Ideally, we would want to see consistency in both the percentage of patients and practices that meet our study criteria, as this would preserve the representativeness of the CPRD dataset. We observe that the South East Coast has both the most practices and patients that meet the criteria for our study. On the other hand, the East Midlands does not have any patients that qualify for the study. This is because, in our data extract (March 2016 snapshot), there has been no data collected from a practice in the East Midlands since 2014. Despite these regions, the average percentage of patients included in the study is 38.8% with an interquartile range of 19.2-41.0%. Similarly, the percentage of practices included in the study is 37.4% with an interquartile range of 19.5-45.2%. This indicates reasonable consistency across the dataset. Furthermore, the consistency must be weighed against other factors, such as data quality. For this reason, we feel the slight compromise in consistency resulting from the exclusion of practices in the East Midlands and elsewhere, due to a lack of recent data, is passable.

References

1. Campbell J, Dedman D, Eaton S, Gallagher A, Williams T. Is the CPRD GOLD population comparable to the UK population. Pharmacoepidemiol Drug Saf. 2013;22(Suppl 1):280–1.

2. Herrett E, Gallagher AM, Bhaskaran K, Forbes H, Mathur R, van Staa T, et al. Data Resource Profile: Clinical Practice Research Datalink (CPRD). Int J Epidemiol. 2015 Jun;44(3):827–36.
