# Supplementary material for: What is the impact of regulatory guidance and expiry of drug patents on dementia drug prescriptions in England? A trend analysis in the Clinical Practice Research Datalink
Source: Alzheimers Res Ther. 2018 May 29;10:51. doi: 10.1186/s13195-018-0379-6 (PMC5975499; doi:10.1186/s13195-018-0379-6)
Supplement: Supplementary file 2 — Analysis of the sensitivity and specificity of the diagnoses. (PDF 438 kb) [file 13195_2018_379_MOESM2_ESM.pdf]

Additional file 2

Analysis of the sensitivity and specificity of the diagnoses

For this analysis we considered the sensitivity and specificity of the diagnoses (see Table 2 in the manuscript) in the CPRD dataset. Sensitivity indicates whether patients in the CPRD have been 'correctly diagnosed' based on the information in comparator dataset, while specificity indicates whether patients have been 'correctly not diagnosed'. Linked data from the Office of National Statistics (ONS) death registry and the Hospital Episode Statistics (HES) inpatient dataset was used as the comparators. HES outpatient data was excluded from the sensitivity analysis as it is known that less than 5.0% of patients have diagnosis recorded in this dataset. (1) This analysis is restricted to patients from practices in England with linked data, which is available for 29,362 patients out of the 40,202 included in the study (73.0%).

We defined the four terms, necessary for the calculation of sensitivity and specificity, as follows:

- True positive: the patient has the code in the CPRD and in the linked data
- False positive: the patient does not have the code in the CPRD but it is in the linked data
- True negative: the patient does not have the code in the CPRD or the linked data
- False negative: the patient has the code in the CPRD but not in the linked data

The sensitivity and specificity could then be calculated as follows (2):

$$\text{Sensitivity} = \frac{\text{true positives}}{\text{true positives} + \text{false negatives}}$$

$$\text{Specificity} = \frac{\text{true negatives}}{\text{true negatives} + \text{false positives}}$$

Diagnosis definitions in the CPRD are determined by Read codes, whereas both the ONS death registry and the HES inpatient dataset use codes from the International Statistical Classification of Diseases and Related Health Problems (ICD). The ICD is maintained by the World Health Organization (WHO) and is currently in its 10th revision: ICD-10. To use this data, we created ICD-10 code lists that correspond to the Read code lists used for the CPRD data extract. These ICD-10 code lists are available online. (3) ICD-10 and Read codes do not map to each other exactly, with ICD-10 codes generally covering multiple Read codes. As we had been conservative and specific with our approach to the Read codes, we included ICD-10 codes on multiple code lists where appropriate. This helped to ensure the scope of our Read code lists was covered when using the less specific ICD-10 codes. For example, the ICD-10 code 'F03' represents 'Unspecified dementia' and includes the following, many of which refer to diagnoses that are not otherwise specified (NOS):

- Presenile dementia NOS
- Presenile psychosis NOS
- Primary degenerative dementia NOS
- Senile dementia NOS
- Senile dementia, depressed or paranoid type
- Senile psychosis NOS

There are Read codes for each of the above bullet points and for 'Unspecified dementia'. In our Read code lists, we assigned the codes 'Unspecified dementia' and 'Primary degenerative dementia NOS' to the non-specific dementia code list and the remaining codes to the possible Alzheimer's disease code list. We therefore chose to include the ICD-10 code 'F03' on both the non-specific dementia and possible Alzheimer's disease ICD-10 code lists to account for the multiple Read codes it relates to.

Table S2.1 presents the sensitivity and specificity of the diagnoses using the ONS death registry as the comparator. Sensitivity for the diagnosis possible Alzheimer's disease is poor (36.0%), however the other diagnoses perform much better with non-AD and mixed dementias performing the best (80.4%). The specificity of the diagnoses is generally much better across all diagnoses (≥ 57.5%) with the diagnosis probable Alzheimer's disease being the most specific (75.1%). The higher specificity of the code lists reflects our conservative approach to the Read code lists, which are used in combination to determine diagnosis.

Because of this, we expected a lower sensitivity, and this is in line with what we observed. While sensitivity is low for the Alzheimer's disease diagnoses, particularly the possible cases, the larger sample size used in our study (that includes patients without linked data) means we have ample power, even if some patients are missed.

Table S2.1: The sensitivity and specificity of the diagnoses used in the CPRD and ONS datasets.

	Patients in CPRD dataset	Patients in ONS death registry	Sensitivity	Specificity
Probable AD	8069	1863	65.5	75.1
Possible AD	8259	4752	36.0	73.4
Non-AD and mixed dementias	13034	1456	80.4	57.5

AD: Alzheimer's disease; CPRD: Clinical Practice Research Datalink; ONS: Office of National Statistics

Tables S2.2 presents the sensitivity and specificity of the diagnoses using the HES inpatient dataset as the comparator. The general pattern across diagnoses is much the same as that observed in the analysis that used the ONS death registry as the comparator. Both probable Alzheimer's disease and non-AD and mixed dementias have slightly lower sensitivity but higher specificity than the previous analysis. Meanwhile possible Alzheimer's disease has minor improvements in both sensitivity and specificity. This is likely due to the ONS death registry recording diagnoses at the time of, or after, death. Because of this, you might expect possible diagnoses to be less relevant and potentially other forms of evidence to be available to preclude conditions (e.g. through post mortem). Overall, both this table and the previous, indicate variable sensitivity with high specificity of the diagnoses. Encouragingly, the definition of probable Alzheimer's disease, which is used in the main analysis, performs well in both categories.

Table S2.2: The sensitivity and specificity of the diagnoses used in the CPRD and HES datasets.

	Patients in CPRD dataset	Patients in HES inpatient dataset	Sensitivity	Specificity
Probable AD	8069	5007	59.4	79.1
Possible AD	8259	6461	37.3	74.5
Non-AD and mixed dementias	13034	6206	71.6	62.9

AD: Alzheimer's disease; CPRD: Clinical Practice Research Datalink; HES: Hospital Episodes Statistics

References

1. Medicine & Healthcare products Regulatory Agency, National Institute for Health Research, Clinical Practice Research Datalink. Hospital Episode Statistics (HES) Outpatient Care and GOLD Documentation (Set 12). 2016.

2. Lalkhen AG, McCluskey A. Clinical tests: sensitivity and specificity. Contin Educ Anaesth Crit Care Pain. 2008 Dec 1;8(6):221–3.

3. Walker V, Davies N, Kehoe P, Martin R. CPRD codes: ICD-10 equivalent code lists for dementia subtypes. 2017; Available from: https://doi.org/10.5523/bris.2h4rmk9v7pw2k23h7vgf9tx1ea
