# Supplementary material for: What is the impact of regulatory guidance and expiry of drug patents on dementia drug prescriptions in England? A trend analysis in the Clinical Practice Research Datalink
Source: Alzheimers Res Ther. 2018 May 29;10:51. doi: 10.1186/s13195-018-0379-6 (PMC5975499; doi:10.1186/s13195-018-0379-6)
Supplement: Supplementary file 3 — Analysis using alternative diagnoses. (PDF 1728 kb) [file 13195_2018_379_MOESM3_ESM.pdf]

Additional file 3

Analysis using alternative diagnoses

Introduction

The main analysis considers the diagnosis probable Alzheimer's disease. As a sensitivity analysis, we also considered two alternative diagnoses. The first, any Alzheimer's disease, considers patients diagnosed with probable or possible Alzheimer's disease. The second, any dementia, considers patients diagnosed with any form of dementia i.e. probable Alzheimer's disease, possible Alzheimer's disease, non-Alzheimer's disease and mixed dementias. This additional file presents the trend graphs and joinpoint analysis output tables for each combination of diagnosis and drug class.

Note for all Joinpoint analysis output tables

Segments are the period between two join points and are numbered chronologically. Segment 1 begins at the analysis start date (June 1997 for AChE inhibitors and January 2003 for NMDA receptor antagonists) and the remaining segments begin with each joinpoint. Segment 3 ends in December 2015.

Note for all trend graphs

The fixed lines indicate events with the potential to effect prescription rates during the study period. The joinpoints, monthly percent change for each segment and the average monthly percent change for the entire study period are also presented.

List of Abbreviations

AD: Alzheimer's Disease
AChE: AcetylCholinEsterase
AMPC: Average Monthly Percent Change
CPRD: Clinical Practice Research Datalink
HES: Hospital Episode Statistics
MMSE: Mini Mental State Examination
MPC: Monthly Percent Change
NICE: National Institute of health and Care Excellence
NMDA: N-Methyl-D-Aspartate
ONS: Office of National Statistics
QOF: Quality and Outcomes Framework

Probable Alzheimer's disease & AChE inhibitor prescriptions

Figure 2: Indicative graph of AChE inhibitor prescriptions in patients with probable Alzheimer's disease.

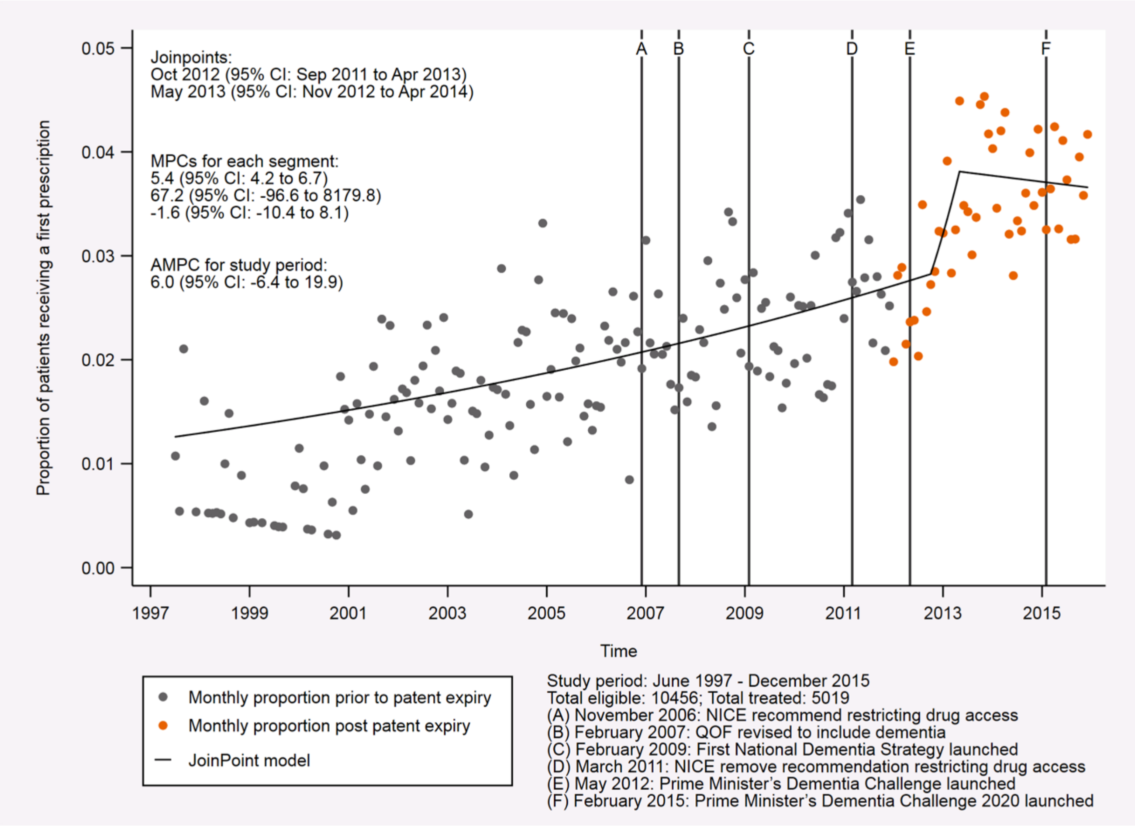

Table S3.1: Joinpoint analysis output for AChE inhibitor prescriptions in patients with probable Alzheimer's disease.

	Segment 1	Segment 2	Segment 3
Joinpoint	Not applicable	October 2012	May 2013
Joinpoint 95% LCL	Not applicable	September 2011	November 2012
Joinpoint 95% UCL	Not applicable	April 2013	April 2014
Intercept estimate	-110.174	-1038.186	28.411
Intercept standard error	12.401	3982.069	95.917
Intercept test statistic	-8.884	-0.261	0.296
Intercept p-value	0.000	0.795	0.767
Slope estimate	0.053	0.514	-0.016
Slope standard error	0.006	1.978	0.048
Slope test statistic	8.576	0.260	-0.331
Slope p-value	0.000	0.795	0.741
Slope change estimate	Not applicable	0.461	-0.530
Slope change standard error	Not applicable	1.978	1.979
Slope change test statistic	Not applicable	0.233	-0.268
Slope change p-value	Not applicable	0.816	0.789

Probable Alzheimer's disease & NMDA receptor antagonist prescriptions

Figure 3: Indicative graph of NMDA receptor antagonist prescriptions in patients with probable Alzheimer's disease.

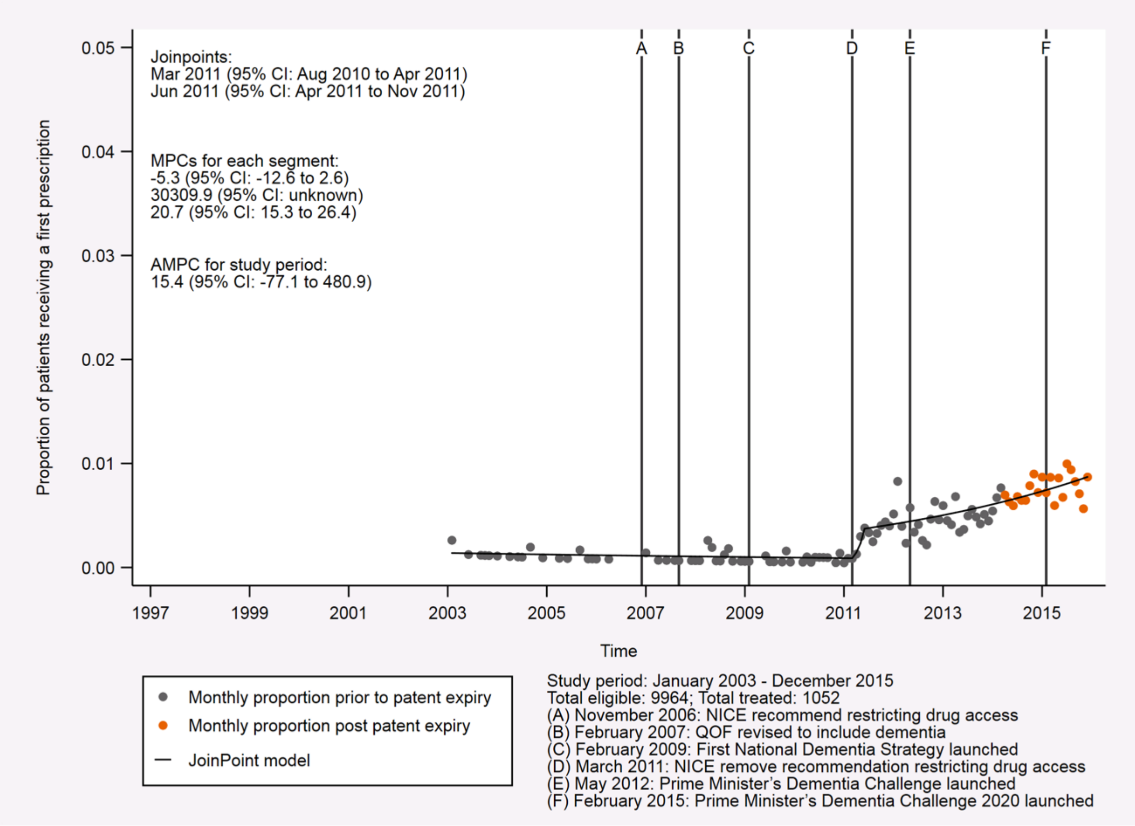

Table S3.2: Joinpoint analysis output for NMDA receptor antagonist prescriptions in patients with probable Alzheimer's disease.

	Segment 1	Segment 2	Segment 3
Joinpoint	Not applicable	March 2011	June 2011
Joinpoint 95% LCL	Not applicable	August 2010	April 2011
Joinpoint 95% UCL	Not applicable	April 2011	November 2011
Intercept estimate	102.447	-11505.572	-384.099
Intercept standard error	81.399	85104.301	46.589
Intercept test statistic	1.259	-0.135	-8.244
Intercept p-value	0.211	0.893	0.000
Slope estimate	-0.054	5.717	0.188
Slope standard error	0.041	42.313	0.023
Slope test statistic	-1.343	0.135	8.136
Slope p-value	0.183	0.893	0.000
Slope change estimate	Not applicable	5.772	-5.529
Slope change standard error	Not applicable	42.314	42.313
Slope change test statistic	Not applicable	0.136	-0.131
Slope change p-value	Not applicable	0.892	0.896

Any Alzheimer's disease & AChE inhibitor prescriptions

Figure S3.1: Indicative graph of AChE inhibitor prescriptions in patients with any Alzheimer's disease.

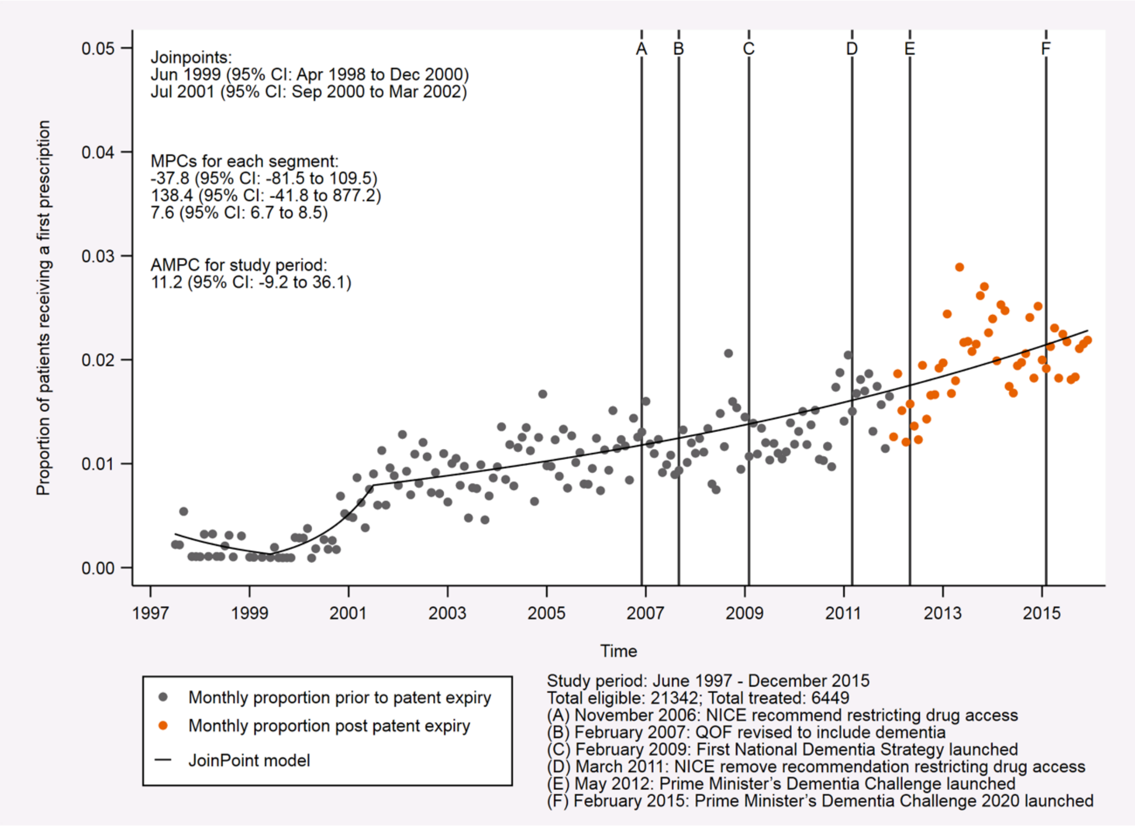

Table S3.3: Joinpoint analysis output for AChE inhibitor prescriptions in patients with any Alzheimer's disease.

	Segment 1	Segment 2	Segment 3
Joinpoint	Not applicable	June 1999	July 2001
Joinpoint 95% LCL	Not applicable	April 1998	September 2000
Joinpoint 95% UCL	Not applicable	December 2000	March 2002
Intercept estimate	943.277	-1743.896	-151.473
Intercept standard error	1230.225	1430.504	8.202
Intercept test statistic	0.767	-1.219	-18.468
Intercept p-value	0.444	0.224	0.000
Slope estimate	-0.475	0.869	0.073
Slope standard error	0.616	0.715	0.004
Slope test statistic	-0.772	1.215	17.965
Slope p-value	0.441	0.226	0.000
Slope change estimate	Not applicable	1.344	-0.796
Slope change standard error	Not applicable	0.944	0.715
Slope change test statistic	Not applicable	1.424	-1.113
Slope change p-value	Not applicable	0.156	0.267

Any Alzheimer's disease & NMDA receptor antagonist prescriptions

Figure S3.2: Indicative graph of NMDA receptor antagonist prescriptions in patients with any Alzheimer's disease.

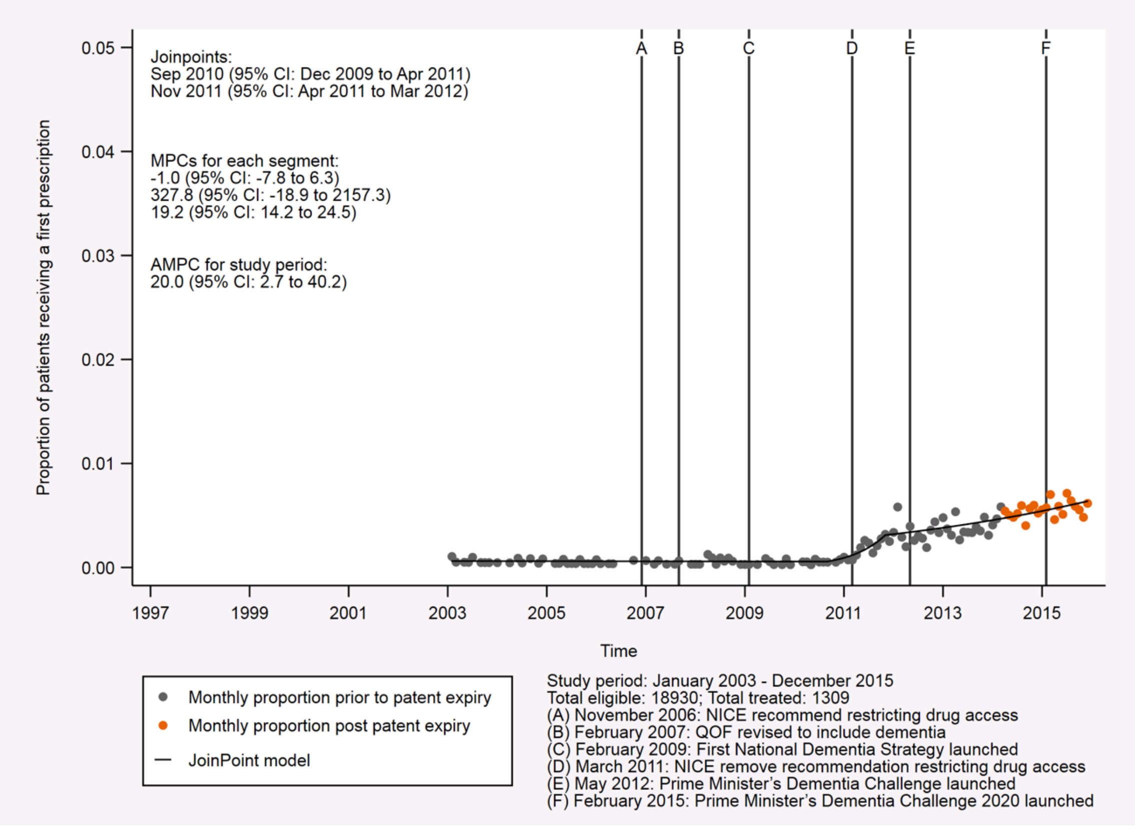

Table S3.4: Joinpoint analysis output for NMDA receptor antagonist prescriptions in patients with any Alzheimer's disease.

	Segment 1	Segment 2	Segment 3
Joinpoint	Not applicable	September 2010	November 2011
Joinpoint 95% LCL	Not applicable	December 2009	April 2011
Joinpoint 95% UCL	Not applicable	April 2011	March 2012
Intercept estimate	12.716	-2930.156	-359.411
Intercept standard error	72.053	1686.414	43.800
Intercept test statistic	0.176	-1.738	-8.206
Intercept p-value	0.860	0.085	0.000
Slope estimate	-0.010	1.454	0.176
Slope standard error	0.036	0.838	0.022
Slope test statistic	-0.280	1.734	8.084
Slope p-value	0.780	0.086	0.000
Slope change estimate	Not applicable	1.464	-1.278
Slope change standard error	Not applicable	0.839	0.839
Slope change test statistic	Not applicable	1.744	-1.523
Slope change p-value	Not applicable	0.084	0.131

Any dementia & AChE inhibitor prescriptions

Figure S3.3: Indicative graph of AChE inhibitor prescriptions in patients with any dementia.

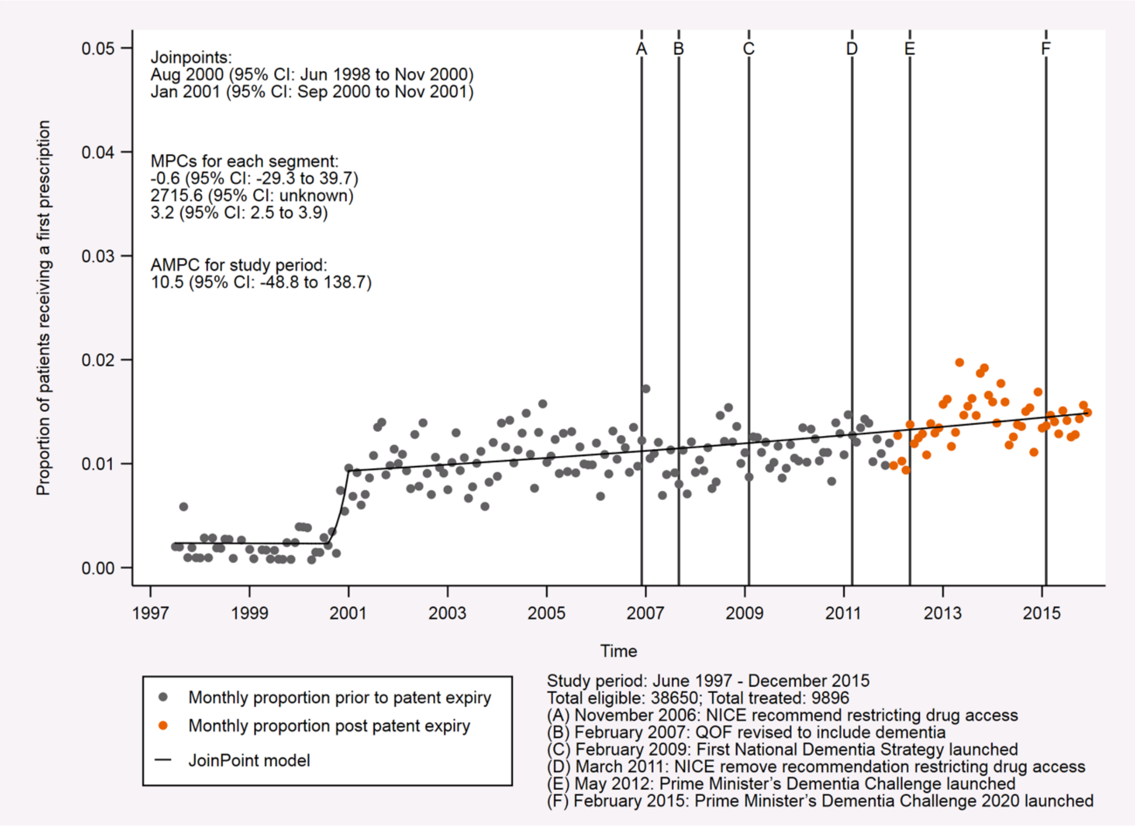

Table S3.5: Joinpoint analysis output for AChE inhibitor prescriptions in patients with any dementia.

	Segment 1	Segment 2	Segment 3
Joinpoint	Not applicable	August 2000	January 2001
Joinpoint 95% LCL	Not applicable	June 1998	September 2000
Joinpoint 95% UCL	Not applicable	November 2000	November 2001
Intercept estimate	6.837	-6683.509	-67.325
Intercept standard error	345.577	34621.871	6.807
Intercept test statistic	0.020	-0.193	-9.890
Intercept p-value	0.984	0.847	0.000
Slope estimate	-0.006	3.338	0.031
Slope standard error	0.173	17.305	0.003
Slope test statistic	-0.037	0.193	9.248
Slope p-value	0.970	0.847	0.000
Slope change estimate	Not applicable	3.344	-3.306
Slope change standard error	Not applicable	17.305	17.305
Slope change test statistic	Not applicable	0.193	-0.191
Slope change p-value	Not applicable	0.847	0.849

Any dementia & NMDA receptor antagonist prescriptions

Figure S3.4: Indicative graph NMDA receptor antagonist prescriptions in patients with any dementia.

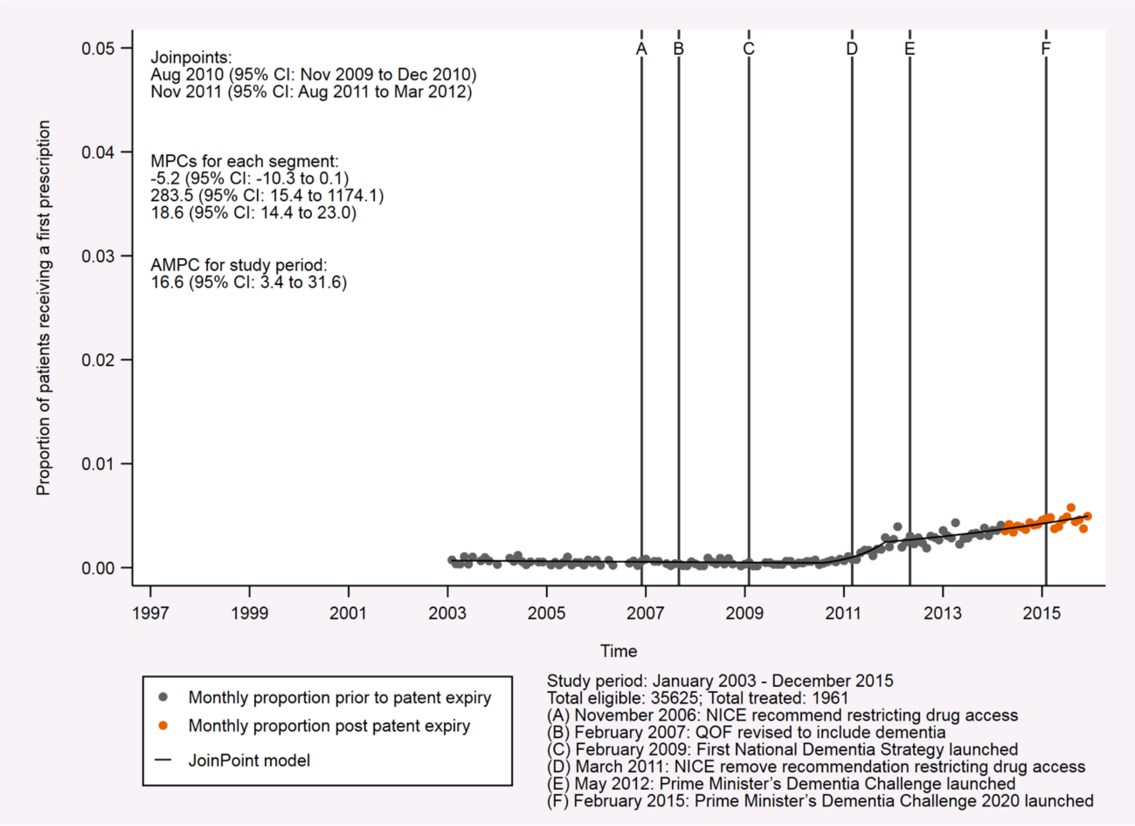

Table S3.6: Joinpoint analysis output for NMDA receptor antagonist prescriptions in patients with any dementia.

	Segment 1	Segment 2	Segment 3
Joinpoint	Not applicable	August 2010	November 2011
Joinpoint 95% LCL	Not applicable	November 2009	August 2011
Joinpoint 95% UCL	Not applicable	December 2010	March 2012
Intercept estimate	100.313	-2710.001	-349.231
Intercept standard error	55.359	1219.196	36.632
Intercept test statistic	1.812	-2.223	-9.534
Intercept p-value	0.073	0.028	0.000
Slope estimate	-0.054	1.344	0.171
Slope standard error	0.028	0.606	0.018
Slope test statistic	-1.947	2.217	9.382
Slope p-value	0.054	0.029	0.000
Slope change estimate	Not applicable	1.398	-1.173
Slope change standard error	Not applicable	0.607	0.606
Slope change test statistic	Not applicable	2.303	-1.935
Slope change p-value	Not applicable	0.023	0.055
